# Cancer heterogeneity and multilayer spatial evolutionary games

**DOI:** 10.1186/s13062-016-0156-z

**Published:** 2016-10-13

**Authors:** Andrzej Świerniak, Michał Krześlak

**Affiliations:** Department of Automatic Control, Silesian University of Technology, ul. Akademicka 16, 44-100 Gliwice, Poland

**Keywords:** Evolutionary games, Biomathematical modelling, Heterogeneity, Cancer, Spatial effects, Sensitivity

## Abstract

**Background:**

Evolutionary game theory (EGT) has been widely used to simulate tumour processes. In almost all studies on EGT models analysis is limited to two or three phenotypes. Our model contains four main phenotypes. Moreover, in a standard approach only heterogeneity of populations is studied, while cancer cells remain homogeneous. A multilayer approach proposed in this paper enables to study heterogeneity of single cells.

**Method:**

In the extended model presented in this paper we consider four strategies (phenotypes) that can arise by mutations. We propose multilayer spatial evolutionary games (MSEG) played on multiple 2D lattices corresponding to the possible phenotypes. It enables simulation and investigation of heterogeneity on the player-level in addition to the population-level. Moreover, it allows to model interactions between arbitrary many phenotypes resulting from the mixture of basic traits.

**Results:**

Different equilibrium points and scenarios (monomorphic and polymorphic populations) have been achieved depending on model parameters and the type of played game. However, there is a possibility of stable quadromorphic population in MSEG games for the same set of parameters like for the mean-field game.

**Conclusion:**

The model assumes an existence of four possible phenotypes (strategies) in the population of cells that make up tumour. Various parameters and relations between cells lead to complex analysis of this model and give diverse results. One of them is a possibility of stable coexistence of different tumour cells within the population, representing almost arbitrary mixture of the basic phenotypes.

**Reviewers:**

This article was reviewed by Tomasz Lipniacki, Urszula Ledzewicz and Jacek Banasiak.

## Background

Heterogeneity of malignant tumour populations has become one of the most often discussed issues related to cancer development and progression. One approach to understand and analyse heterogeneity of cancer cell population employs evolutionary game theory initiated by John Maynard Smith’s works (e.g. [1, 2]). It links mathematical tools of the game theory with Darwinian adaptation and species evolution. In this case players are representatives of the population, and their strategies (phenotypes) are determined genetically. Payoffs in such games represent measures of fitness for the given phenotypes as a result of their interaction.

**Table 1:** Proposed pay-off matrix

Strategies	A (growth factor-producing)	P (cytotoxin-producing)	Q (cytotoxin-resistant)	R (neutral)
A	1-i + j	1 + j-e + g	1 + j-h	1 + j
P	1-i + j-f	1-f-e + g	1-h	1-f
Q	1-i + j	1-e	1-h	1
R	1-i + j	1-e + g	1-h	1

The individuals compete or cooperate with each other to obtain better access to food supplies, life space or spouses. The standard example and the fundamental evolutionary model is Hawk and Dove game. This game, studied by Maynard Smith [2], is a finite nonzero sum game and assumes that the population contains two phenotypes: aggressive (non-outgoing) and compliant. Population members fight for a resource V which affects the reproductive success, but they can also suffer wounds C (the phenotype called Hawk that always takes a fight). This model has been developed into a number of generalizations including spatial effects, evolution in time or other strategies e.g. a legalist strategy (a phenotype can switch between strategies depending on a situation) [3].

Application of the evolutionary game theory to the mathematical modelling of carcinogenesis process is based on the following assertions:

- in an organism, cells compete for nutrients, with different kinds of cells being players in the game
- mutations (observed in tumour cells) may occur during cell division
- an advantage of tumour cells over healthy ones is a signature of cancer.

One of the first works, where the evolutionary game theory was used to model the interaction behaviour of tumour cells, was presented by Tomlinson [4]. The author proposed the model, where one of the phenotypes attempts to gain an advantage by producing cytotoxic substances. Results show that actively harming neighbouring cells may lead to dominance of the local population by the tumour cells. This study has triggered a series of other papers, where evolutionary game theory has been applied to describe phenomena of tumour creation by mechanisms of avoidance of apoptosis [5], creation of additional capillaries as a result of angiogenesis [5, 6], and development of capabilities of invading other tissues and metastasis [7, 8], and many others. On the other hand, game theory models show only single phenomena occurring in a very complicated process of cancer evolution. Dynamics of the system under consideration, which can be analysed by replicator dynamics equations [9, 10]. In this approach the dynamics of the strategy profile for a population is defined by the Malthusian type growth described by the set of ODE’s.

In our paper [11] we have extended the idea described above to study a model of radiation induced bystander effect in a cell population and to predict its dynamics using replicator equations.

Unfortunately, in almost all studies on EGT models, analysis is limited to two or three phenotypes. The exception is our paper [12] in which interactions between four different phenotypes of cells are illustrated using three-dimensional simplexes and time courses. As far as we know, the only other work which includes four phenotypes is [13]. However, instead of studying different equilibrium points between phenotypes and their dynamics, the authors have analysed only final results (different subpopulations) with respect to changes of fitness parameters.

It is important to notice that dimension of replicator dynamics equations in the case of three phenotypes is equal to two which means that complex dynamical behaviours, typical for nonlinear dynamics should be absent. In our opinion it is one of major disadvantages of the small number of considered strategies. An important finding is that a four-phenotype model implies third-order dynamics of replication which enables existence of complex dynamical behaviours including strange attractors. This may be an important hallmark of evolutionary game theory analysis. To illustrate advantages of our approach to analysis of increasing number of strategies, let us consider the model which combines two classical models of Tomlinson ([4, 5]).

There are several ways to resolve evolutionary stable games. One possibility is to solve replicator dynamics equations for mean-field games. Alternatively one can apply cellular automata for spatial evolutionary games. Even though spatial games include another factor (i.e. space) that brings the evolutionary games methods closer to biological phenomena, still the cells are considered to be homogeneous, i.e. in the game theory terms individual cell can play only one strategy. Spatial games incorporating heterogeneity of the cells proposed by us in [14], are called multilayer spatial evolutionary games (MSEG).

## Methods

An equilibrium in the evolutionary games is defined by an evolutionary stable strategy (ESS [1, 15]). It defines a phenotype, which is resistant to an inflow of other phenotypes (resulting from a mutation or environmental migration) and it cannot be repressed by them. However, a reverse situation is possible, evolutionary stable strategy can stay or even dominate population as an inflow mutant. The phenotypes play the role of pure strategies in standard non-cooperative games, the evolutionary strategies are frequencies of individuals in population (so called strategy profiles) representing these phenotypes and in this sense are analogues of mixed strategies. In addition ESS is always Nash equilibrium (in mixed strategies), but reverse implication is generally not true [3]. There are also other differences. In evolutionary games, strategies are genetically programmed and they cannot be changed and a game structure is not clear. In the classical game theory based on Nash equilibrium players know the game structure and rules, and the game (in its repeated form [16]) is played many times in the same conditions, while ESS results rather from the iterated game with varying players frequencies in passing generations.

Moreover, the Nash strategies are the results of rational analysis, while evolutionary strategies are rather due to behaviour shaped through natural selection. The good illustration of this difference is the famous Haldane sentence: *I would jump into a river to save two brothers or eight cousins* [2].

More precisely, the ESS has two properties:

1. It is a mixed Nash strategy
2. It is stable

In the standard game theory the non-zero sum two-person game in normal form is represented by two payoff matrices thus it is also called a bimatrix game. In the evolutionary games the payoffs for players are well defined by a single matrix. Players may use different strategies, but there is no differentiation between them (like strength, age etc.).

Replicator dynamics is one way to resolve evolutionary stable games. It represents the so called mean-field approach. Another technique which enables study of allocation of players is called spatial evolutionary game. It combines the evolutionary game theory with machinery of cellular automata or agent based modelling. In this case is a local players’ position with specific strategies and different ways of performance very important. To our knowledge the first application of spatial game solutions in cancer modelling has been presented by Bach et al. [17] as a development of angiogenic games [5]. Spatial version of the motility/evasion game is presented in [18]. Many works demonstrate that the spatial modelling discloses altruistic and cooperative strategies, and strong discrepancies when compared to the mean-field models (e.g. [19]).

The basic distinctions between mean-field and spatial models is lack of perfect mixing; intercellular interactions are dependent on local population arrangement. While mean-field models are rather simplistic descriptions of carcinogenesis, spatial models, based on cellular automata, constitute the next step to discover new behaviours among cells and give different results than mean-field models. Recently, spatial games have become very popular, nevertheless it should be remembered that their origin is the use of cellular automata by such pioneers as von Neumann [20] in conjunction with the classical theory of games. Mansury and co-workers [8, 21] use the term agent-based modelling to focus on the fact that in such models the smallest unit of observation is the individual tumour cell rather than the entire neoplasm. In our research we follow the line of reasoning presented by Bach et al. [17], where spatial tool used in modelling of carcinogenesis is most suited to our expectations. Some preliminary results for “bystander games” have been discussed in [22].

Similarly to non-spatial games, the spatial ones are also iterated. Game is played on a lattice forming torus, and every competition resulting in a tie is settled randomly.

Passing transient generations we proceed according to the following steps [17]:

- payoff updating - sum of local fitness in a neighbourhood.
- cell mortality - removing a certain number of players.
- reproduction by competition - defining which of the cells (with respect to their the strategies) will appear on an empty place.

In [17] three ways of cell mortality are presented:

- synchronous updating - all the cells die simultaneously and they are replaced according to the strategy of their neighbours in the previous iteration (before dying).
- asynchronous updating - in each generation a single cell, chosen at random, dies and is replaced.
- semi-synchronous updating – the probability of individual cellular mortality is equal to 0.1. So in one generation 10 % of players are deleted from the lattice.

In this paper we are using mainly semi-synchronous updating; this method enables modelling situations that are biologically more realistic. Furthermore, simulations show that synchronous updating assumes a global controller of the system, while asynchronous updating implies that vanishing of small cells clusters is impossible.

The initial lattice is the same for all simulations, but it has been generated randomly to avoid initial clusters. The size of the lattice is 30x30 (contains 900 cells). Moreover, since in our approach each cell is defined by multiple phenotypes, the lattice has another dimension, the size of which equals to the number of basic phenotypes used in the simulation (i.e. 30x30x4). From the point of view of the individual cell and their neighbours the lattice has two dimensions, but due to its heterogeneity the game is played on multiple layers representing separate phenotypes, but connected with each other by the particular cell. This is why we propose to call it a multilayer evolutionary game.

Reproduction of removed players (killed cells) is the next step in the algorithm. It is understood as the way in which empty place after the cell death is invaded by its neighbours. In [17] two kinds of reproduction were proposed:

- a deterministic one – in the competition for an empty place the winner is the strongest player (with highest local adaptation – sum of eight scores from cell-cell interaction)
- a probabilistic one – values of fitness (sum of the values from pay-off matrix) for each player are divided by the total score in their neighbourhood. This local competition, with an appropriate fitness and location, allows cells strategies with lower fitness, but in better location and locally superior in numbers, to predominate in population.

In our opinion, deterministic reproduction is justified when we consider direct interaction of cells, while probabilistic one is more appropriate if the interaction results from signal transduction between cells directly exposed to some external stresses and their neighbours not exposed directly. In other words the probabilistic reproduction is appropriate to model the bystander effect. It seems that in the latter case results of interaction are more “social” than in the former case.

In [17] neighbourhood size is defined in the von Neumann sense (4 neighbours of the cell are taken into account). Other possibilities include the so called Moore neighbourhood (8 neighbours), which is used in our simulations, or extended Moore neighbourhood (24 neighbours).

Results from spatial modelling show that they may be different than mean-field results based on replicator dynamics. Developing spatial model involves enormous range of parameterization possibilities of how to play the game (way of reproduction, deleting players, type of neighbourhood, restriction of lattice, players’ location, size of lattice, initial conditions). Therefore, results of replicator equations are less dependent on initial frequency and are independent of a chosen way of the allocation.

Spatial games show that cooperation and forming common cells clusters are possible. Moreover, this class of models may better describe some phenomena, however they are not completely deterministic models. In reproduction stage and during ties some random effects are shown. The case of a single player surrounded by other players with different strategies is a very good example. According to the payoff matrix evolutionary stable strategy is a strategy of single player. If so, with some amount of luck and mortality of surrounding players, it has a chance to dominate the population.

In the spatial evolutionary games it is also much easier than in the mean-field games to introduce new phenotypes and increase the dimension of the space of strategies.

### Multilayer spatial evolutionary games

The main assumption of the spatial games presented in [17] is that each cell on the lattice is represented by a player following only one strategy. The local payoff for each player is the sum of payoffs due to interactions (according to the payoff matrix) with cells in the neighbourhood. We will refer to this approach as the classical one, or SEGT. Cells on the spatial lattice can also be considered as heterogeneous (instead of homogeneous), so that each particular player may contain mixed phenotypes. Spatial games of the type proposed by us in [14], are called mixed (multilayer) spatial evolutionary games (MSEG). It is important to mention the definition of the phenotype, which is the set of traits or characteristics of an organism [23]. This possibility seems to be especially attractive if stem cells are considered. In this case the strategy played by the cell is almost arbitrary depending on a number of unknown environmental conditions. The choice of a particular strategy may result in cell differentiation and escape to the population of differentiated cells. Alternatively, the cell may retain its stemness. Hence, in MSEG different degrees of playing a particular strategy are treated as different characteristics that define different phenotypes. It may happen that within the population all of the players have diverse phenotypes (which probably better describes biological phenomena). For the sake of simplicity and following the way of reasoning from SEGT, those strategies and traits still correspond to the phenotypes and a general, collective point of view is defined as a player’s phenotypic composition. In fact, the game is performed on a multidimensional lattice (dependent on the number of defined phenotypes in the model, see section: Methods), where each layer represents a particular phenotype (as the frequency of occurrence) of the player. Because of that we propose to call this type of processes multilayer spatial evolutionary games. For the computation of the local adaptation, the sum of the payoffs between each phenotype (within two players) multiplied by their rate of occurrence is calculated first. The second step is the summing of these values for each player in the neighbourhood.

As in SEGT, in every single iteration one global algorithm is used on the lattice, forming a torus. The payoff updating step has been already discussed in general while introducing SEGT and MSEG. More detailed description\is provided further in the text, together with the particular model analysis. The next stage is accounting the cell mortality and in this paper semi-synchronous type is used (10 % of the cells from the lattice are chosen to play this role).

Two kinds of reproduction (deterministic, probabilistic) can also be easily applied for games of this type. A different approach for the player interpretation (polyphenotypic description) allows, however, to create and use other reproductions:

- weighted mean of the strongest players – in accordance with the players’ payoffs, the weighted mean from phenotypes is computed for the players with the highest scores.
- weighted mean of the best interval – players are divided into intervals in accordance with their payoffs. The weighted mean is computed only for the players from the best interval.

Yet another difference between SEGT and MSEG is that the tie (when payoffs are equal) for the former is settled randomly, while for the latter the average between phenotypic compositions is computed. Spatial games are complex due to the vast amount of different methods and parameters.

### Four phenotype model of interaction between tumour cells

The model (Table 1) under consideration contains four different strategies/phenotypes of cells (in order to reduce a number of symbols, phenotypes and their frequencies are denoted by the same symbols):

1. The cell produces the growth factor for its own and all neighbours benefit, for example transforming growth factor-beta TGF-β (we denote frequency of these cells by A);
2. The cell produces a cytotoxic substance against nearby cells, for example cytotoxic lymphocytes (frequency = P);
3. The cell is resistant to the cytotoxic substance, for example cells resistant to cytotoxic lymphocytes (frequency = Q);
4. The strategy which shall be considered as a baseline: the cell does not produce the cytotoxic substance, nor is resistance to it, or growth factor (frequency = R);

This model may be used to study interactions between different cells’ strategies existing in two different models. In terms of tumour cells the sum of A-type (growth factor-producing) and P-type (cytotoxic) may be considered, since Q-type (cytotoxin-resistant) does not make any damage to other cells and R-type is neutral. On the other hand A-type could be considered as cells responsible for immune system, so then P and Q-type shall be tumour cells. In general, the model represents the consequence of interactions between diverse cells’ phenotypes and feasible stable coexistence.

**Table:** 

parameter	description	value range
j	represents the profit of cell contact with growth factors	0–1
i	represents the cost of producing the growth factors	0–1
f	represents the disadvantage of being affected by cytotoxin	0–1
e	represents the cost of producing cytotoxins	0–1
g	represents the profit gained after having subjected another cell to the cytotoxin	0–1
h	represents the cost of resistance to cytotoxin	0–1

To achieve quadruple equilibrium (all phenotypes exist in the final population) the parameters should satisfy some relations resulting from the fact that each expected frequency has to be constrained to the values between 0 and 1. If they are violated, the results may lead to points that indicate other than quadromorphic populations. The equilibrium point could be either an attractor or a repeller and the population itself may be unstable.

## Results

Vast number of parameters and four phenotypes cause that analysis of the model is not as trivial as in the case of two separate models. To check the feasibilities of the model’s final states we present them as functions of two parameters.

**Fig. 1:**
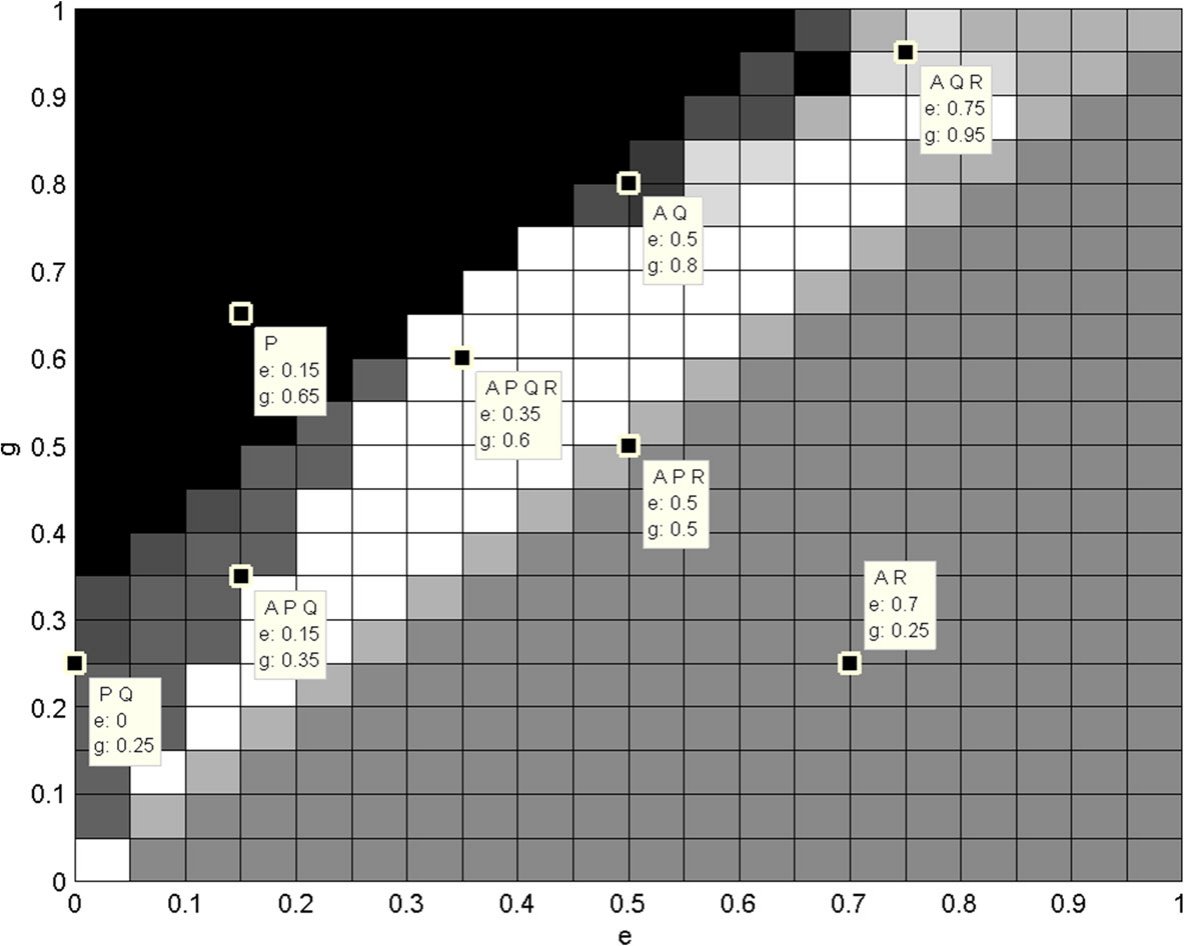
Different subpopulations in accordance to changing parameters. Different subpopulations (represented by shades of grey) in accordance to changing parameters: changing e and g with constant i = 0.3, j = 0.4, h = 0.1, f = 0.4. Some sample points with concrete e and g values and resulting subpopulation are shown

**Fig. 2:**
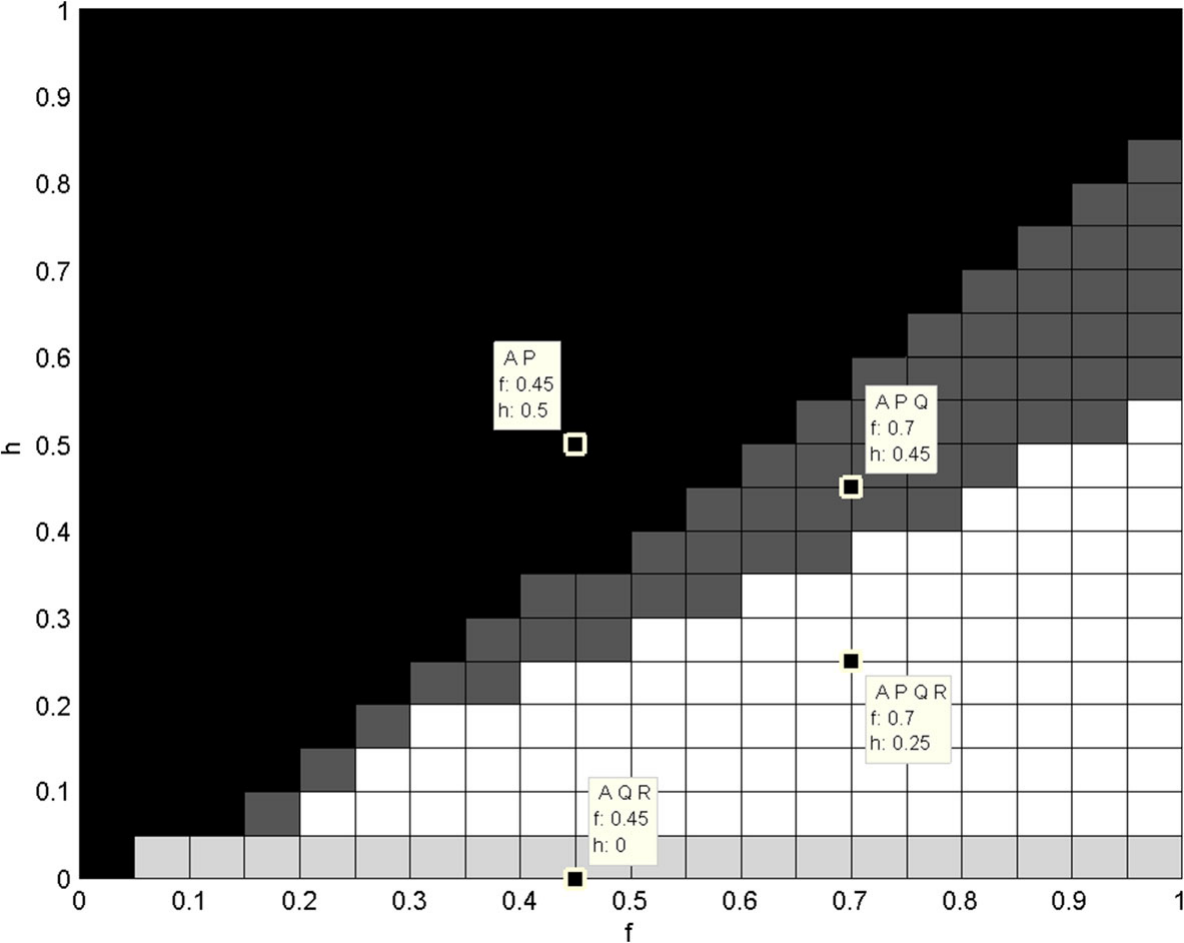
Different subpopulations in accordance to changing parameters. Different subpopulations (represented by shades of grey) in accordance to changing parameters: changing h and f with constant i = 0.3, j = 0.4, e = 0.3, g = 0.4. Some sample points with concrete h and f values and resulting subpopulation are shown

Figures 1 and 2 show that different monomorphic and polymorphic populations may be achieved for various values of parameters. The disadvantage of this approach is that the dynamics and the exact ratios of phenotypes are not shown. Moreover the simulations were performed only for one set of initial frequencies (in this case uniformly distributed). Some basic dependencies may be seen at first glance. For example, if g is smaller than e, then only A and R-cells survive in the population. So, when the profit gained after having subjected another cell to the cytotoxin (g) is not sufficient comparing with cost of cytotoxin productions (e), then P-cells (cytotoxin-producing) are worst adjusted than the rest of the types. At the same time, Q-cells (cytotoxin-resistant), as an evolutionary response to the cytotoxins producers, also lose their advantage in the population. When e equals g then P-cells appear in the population, since their adjustment is the same as the R-cells (neutral). Increasing g leads to different populations, even the quadromorphic one. Then when g is greater than e + 0.35 the population is monomorphic and dominated by P-cells. So when profits are big enough then cytotoxin-producing cells repress other cells from the population. However, it is not clear why the threshold value equals e + 0.35, not any other value.

The second simulation has been performed for changes of h and f. Similarly, as for the previous simulation some threshold values form regions where different polymorphic populations appear: A and P, then when f rises Q-cells stay in population and then R-cells. As can be seen, for this set of parameters usually phenotypes A and P stay in population. The exception is when h = 0, in which case P is repressed from the population due to evolutionary correlation with Q type adaptation.

The results are sensitive to the small changes of the parameter values. It is a matter of changing a value just by 0.1 to achieve different populations in terms of existence of different phenotypes, different evolutionary stable states or even unstable states (i.e. oscillations).

**Fig. 3:**
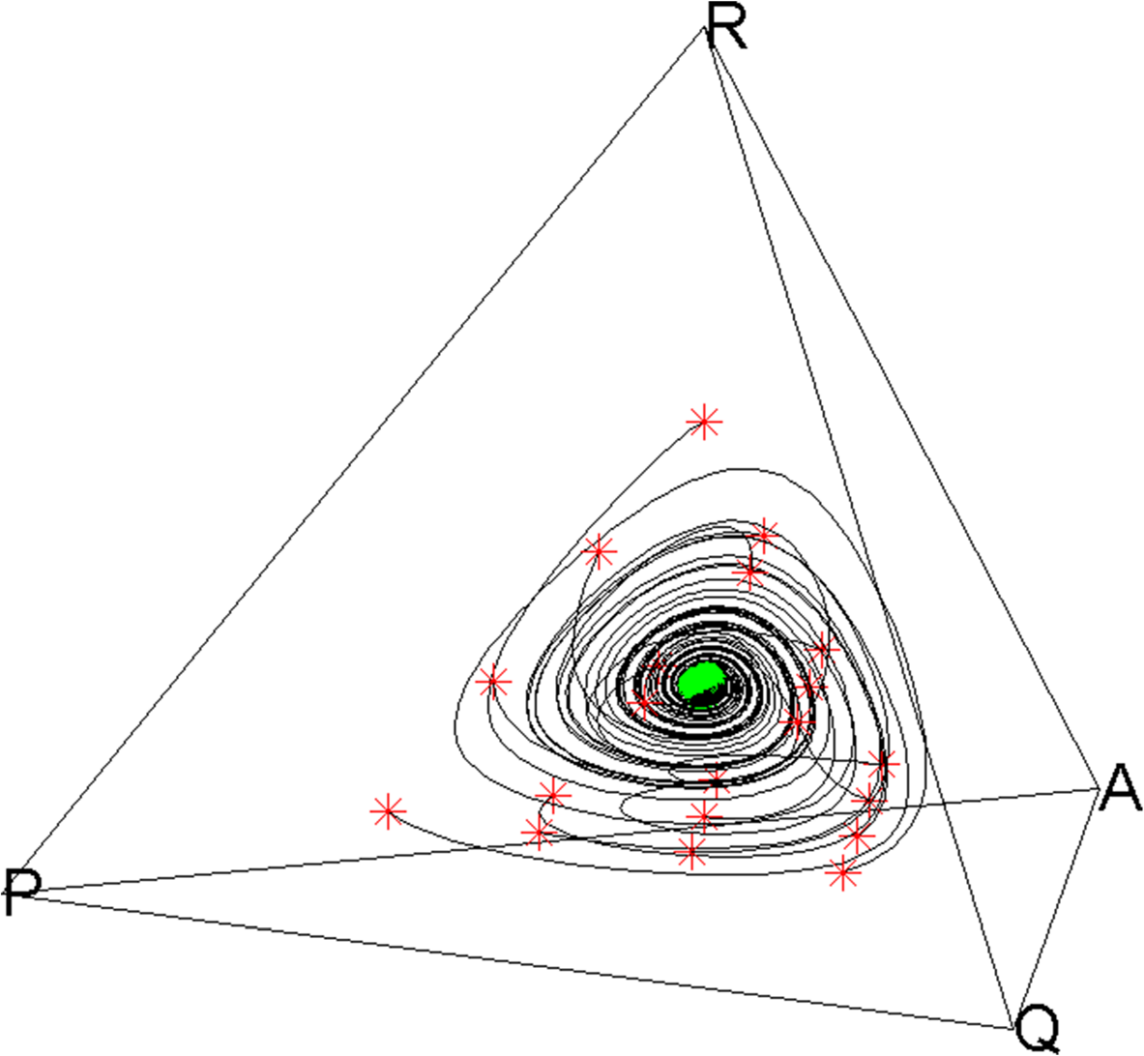
Mean-field results (simplex) for i = 0.3, j = 0.4, f = 0.4, g = 0.4, e = 0.3, h = 0.1. Red asterisks refer to different starting points (initial frequencies of occurrences). Green point refer to the evolutionary stable state

**Fig. 4:**
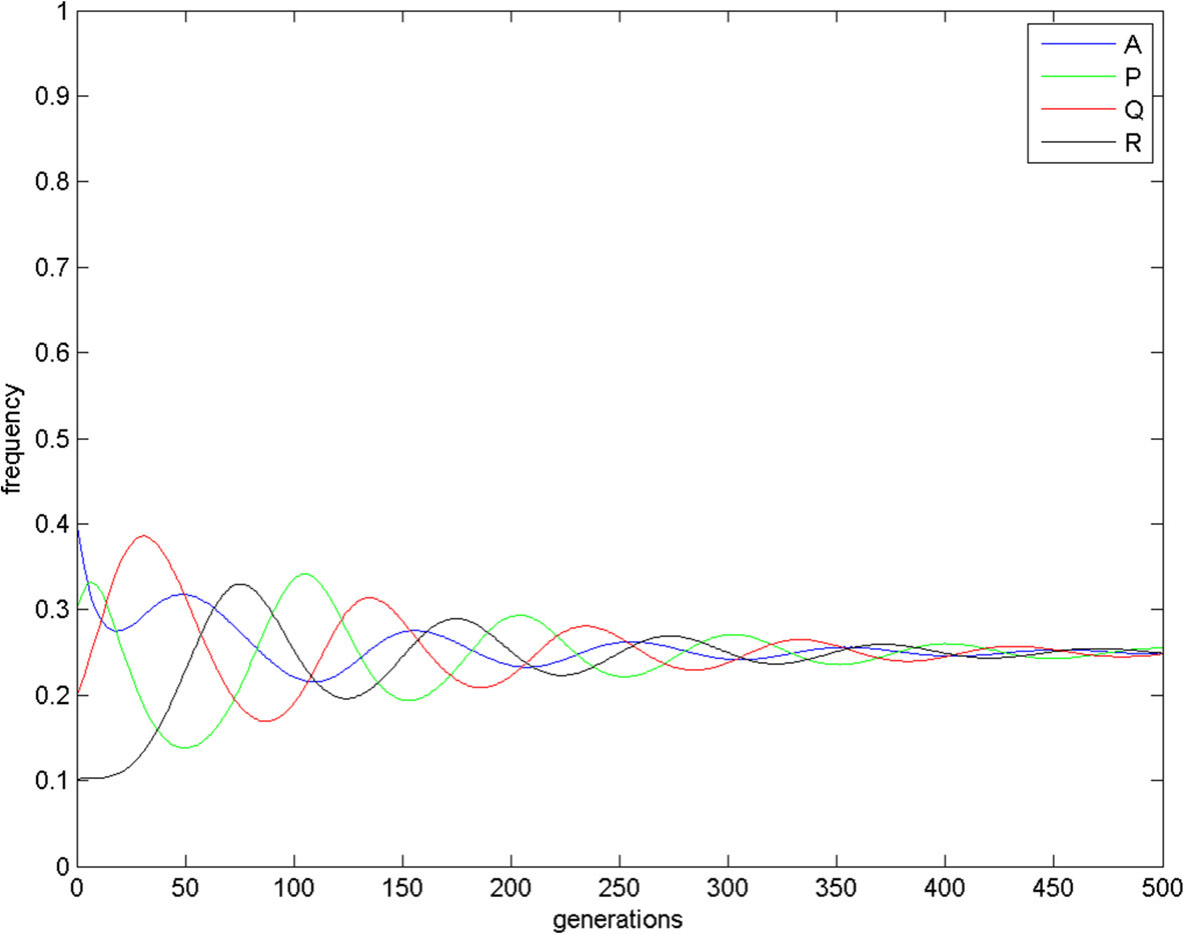
Mean-field results (time chart) for i = 0.3, j = 0.4, f = 0.4, g = 0.4, e = 0.3, h = 0.1

Due to immense amount of different results and combinations of the parameters, we discuss only the case when the population is quadromorphic. The EGT analysis (the mean field model – replicator dynamics) (Figs. 3 and 4) shows that steady state is achieved after some decreasing oscillations and the phenotypes coexist in the population.

**Fig. 5:**
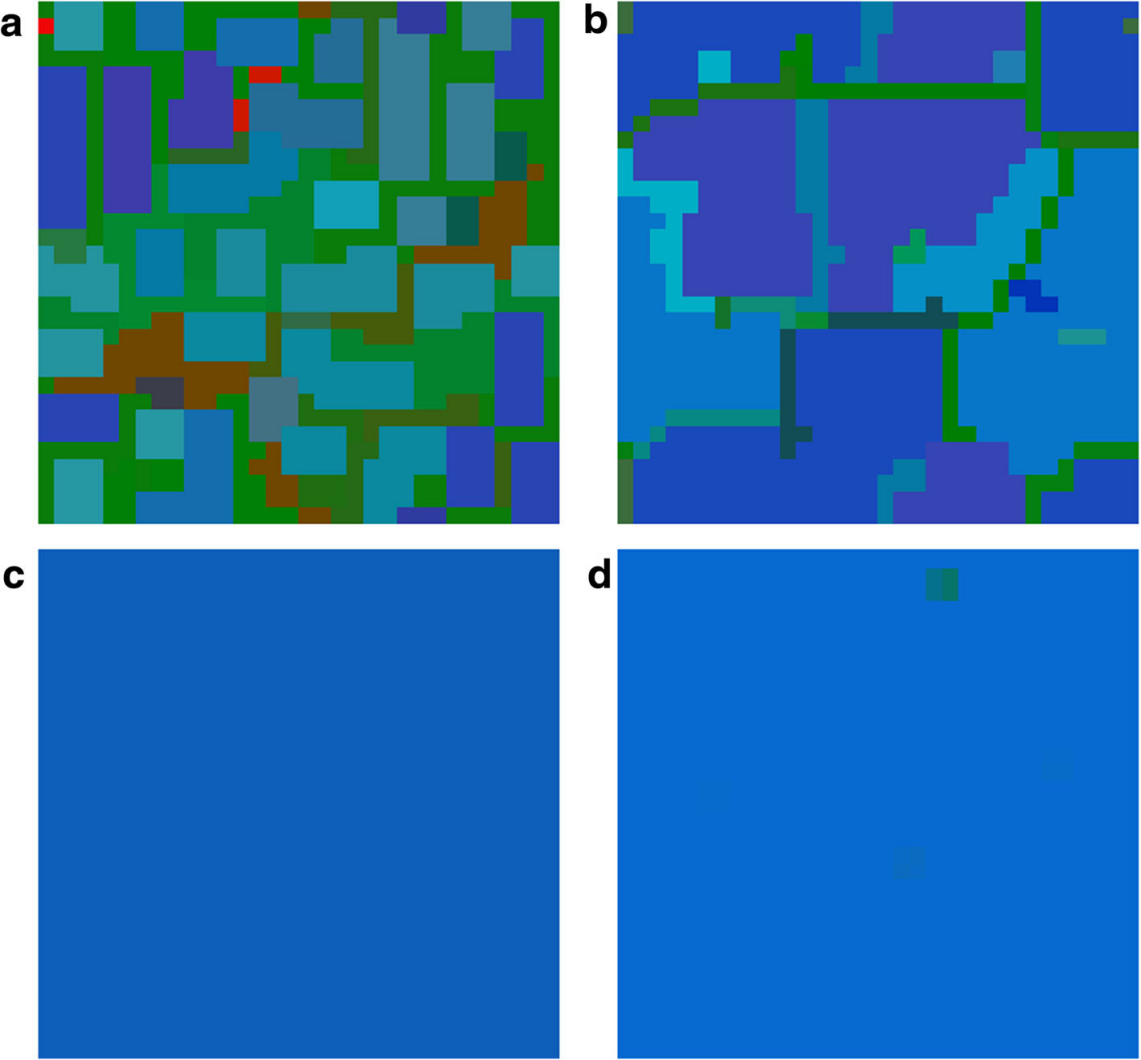
MSEG results (spatial lattice) for i = 0.3, j = 0.4, f = 0.4, g = 0.4, e = 0.3, h = 0.1. **a** probabilistic: A = 0.15, P = 0.40, Q = 0.13, R = 0.32; **b** deterministic: A = 0.36, P = 0.13, Q = 0.10, R = 0.41; **c** weighted mean, best cells 3: A = 0.41, P = 0.12, Q = 0.05, R = 0.42; **d** weighted mean, intervals 5: A = 0.42, P = 0.02, Q = 0.02, R = 0.54. Each phenotype is represented by a different colour (the same as for EGT, see Fig. 4), due to mixed phenotypes for one cells colours are also mixed accordingly

**Fig. 6:**
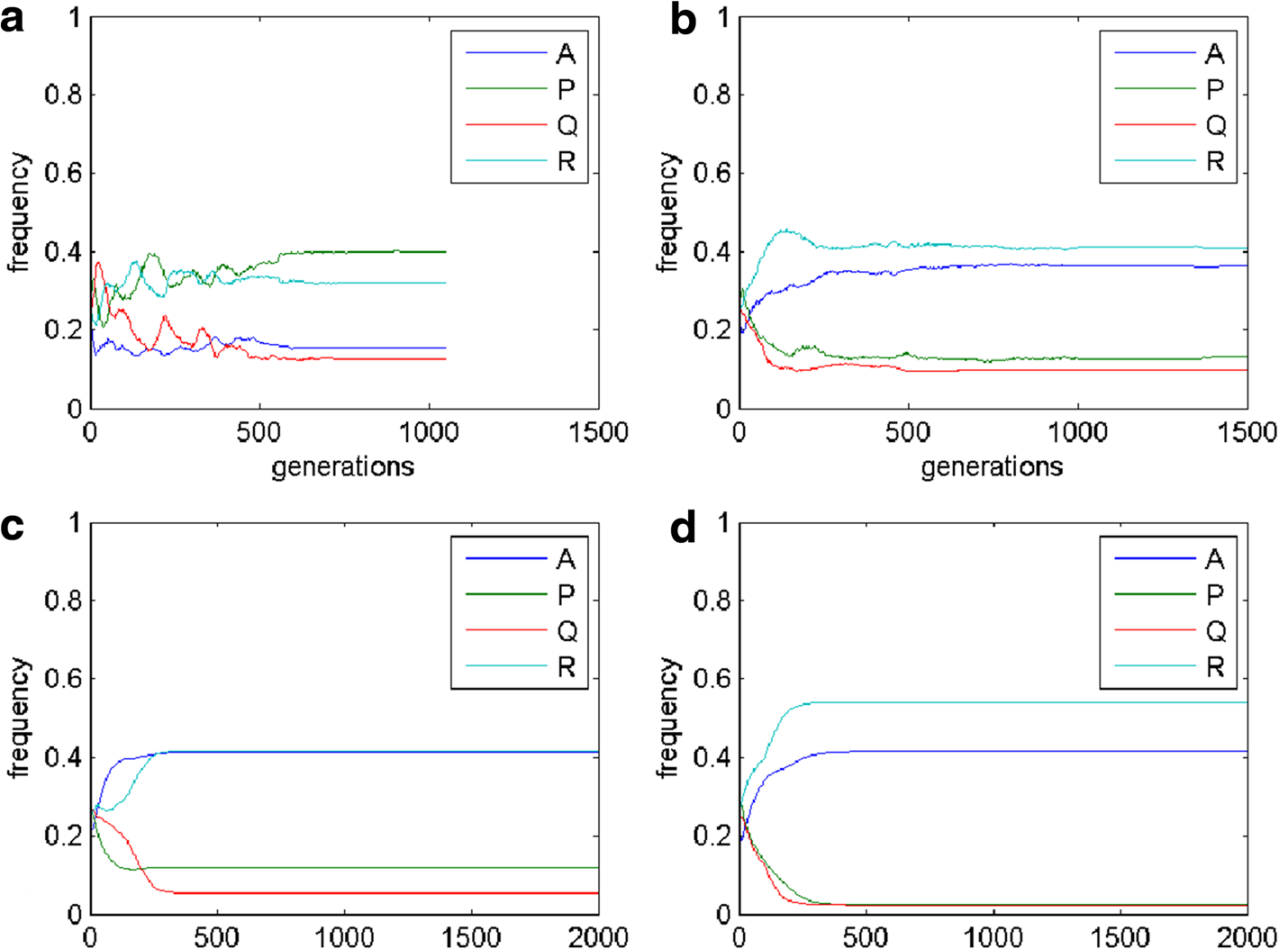
MSEG results (time chart) for i = 0.3, j = 0.4, f = 0.4, g = 0.4, e = 0.3, h = 0.1. **a** probabilistic: A = 0.15, P = 0.40, Q = 0.13, R = 0.32; **b** deterministic: A = 0.36, P = 0.13, Q = 0.10, R = 0.41; **c** weighted mean, best cells 3: A = 0.41, P = 0.12, Q = 0.05, R = 0.42; **d** weighted mean, intervals 5: A = 0.42, P = 0.02, Q = 0.02, R = 0.54

There is a possibility of stable quadromorphic population in MSEG games for the same set of parameters as in the mean-field game (Figs. 5 and 6). In the case of the probabilistic reproduction, the stable state is achieved after some initial oscillations, where the domination of P and R phenotypes appear. Deterministic reproduction gives the domination of A and R phenotypes. Interesting structures of the cells can be found on the lattice, where cells having their phenotype composition dominated by A (navy blue colour) and R (light blue colour) are surrounded by thin “lines” of P phenotype (green colour). For the reproductions based on the weighted mean the same phenotypes prevail in the population, however the phenotype consistency on the lattice is equally spread (averaged), that is the major trend in this kind of reproduction.

**Fig. 7:**
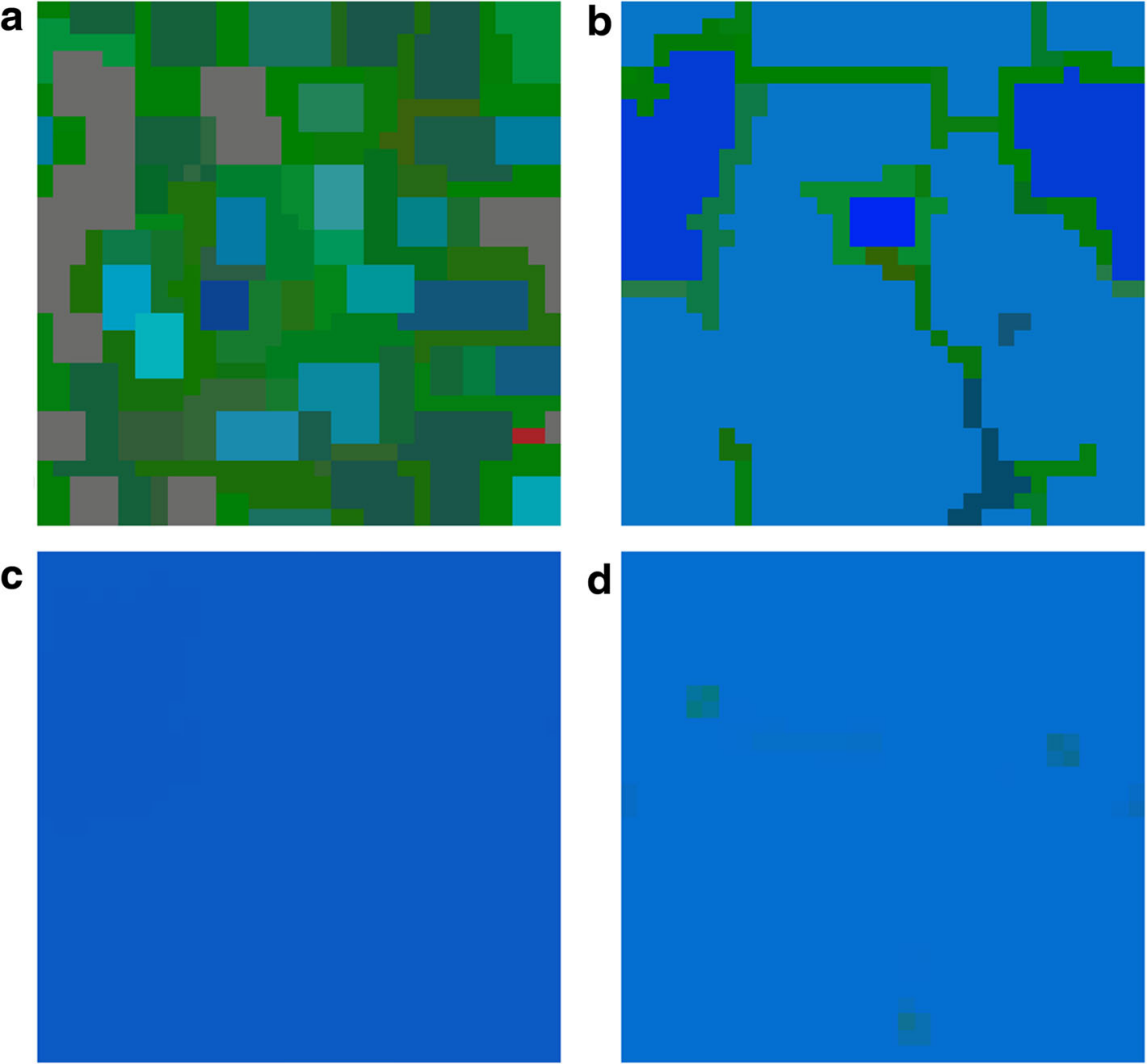
MSEG results (spatial lattice) for i = 0.3, j = 0.4, f = 0.4, g = 0.4, e = 0.3, h = 0.2. **a** probabilistic: A = 0.10, P = 0.55, Q = 0.12, R = 0.23; **b** deterministic: A = 0.34, P = 0.17, Q = 0.03, R = 0.46; **c** weighted mean, best cells 3: A = 0.45, P = 0.08, Q = 0.05, R = 0.42; **d** weighted mean, intervals 5: A = 0.40, P = 0.02, Q = 0.02, R = 0.56

**Fig. 8:**
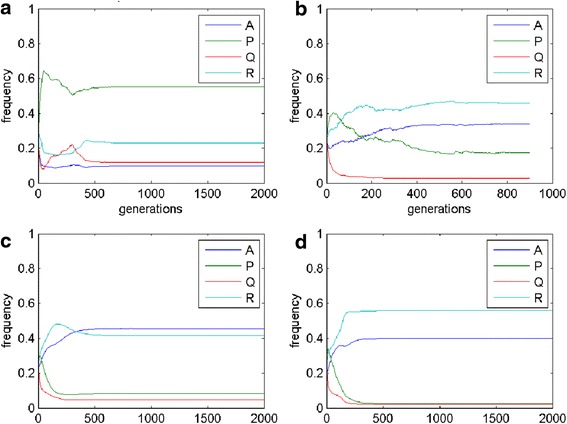
MSEG results (time chart) for i = 0.3, j = 0.4, f = 0.4, g = 0.4, e = 0.3, h = 0.2. **a** probabilistic: A = 0.10, P = 0.55, Q = 0.12, R = 0.23; **b** deterministic: A = 0.34, P = 0.17, Q = 0.03, R = 0.46; **c** weighted mean, best cells 3: A = 0.45, P = 0.08, Q = 0.05, R = 0.42; **d** weighted mean, intervals 5: A = 0.40, P = 0.02, Q = 0.02, R = 0.56

Increasing h to 0.2 (Figs. 7 and 8) causes that in case of probabilistic reproduction the adaptation and the amount of P cells is increased. A similar effect is visible for deterministic reproduction, though the increase of P cells is performed mainly at the cost of Q cells. Weighted-mean reproductions give the same result as for the previous set of parameters. When the parameter e = 0.4, phenotype R is promoted for all kind of reproductions, but for the weighted mean from the best players (here A cells dominate). In the situation, when i = j in case of the mean-field game, oscillations appear between P, Q and R cells. A similar result can be achieved in MSEG games. The probabilistic reproduction after some initial oscillations reaches stability (coexistence between the same phenotypes as in the mean-field game with domination of P and R type). In the case of the deterministic reproduction the changes between the phenotypes are more dynamic even in the later phases of the population evolution.

**Fig. 9:**
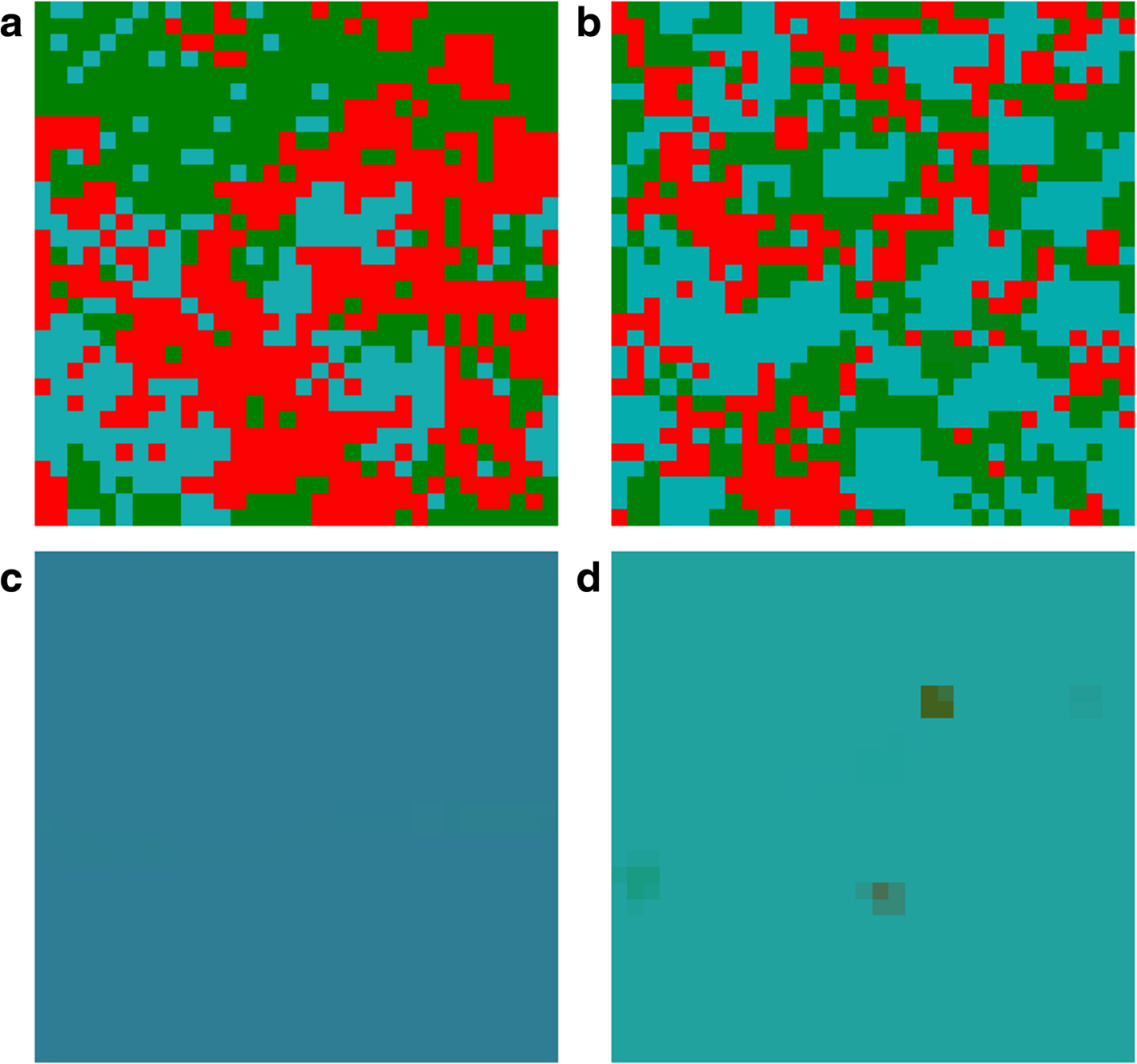
MSEG results (spatial lattice) for i = 0.6, j = 0.4, f = 0.5, g = 0.5, e = 0.3, h = 0.1. **a** probabilistic: A = 0.01, P = 0.36, Q = 0.43, R = 0.20; **b** deterministic: A = 0.02, P = 0.36, Q = 0.26, R = 0.36; **c** weighted mean, best cells 3: A = 0.14, P = 0.09, Q = 0.18, R = 0.59; **d** weighted mean, intervals 5: A = 0.01, P = 0.05, Q = 0.13, R = 0.81

**Fig. 10:**
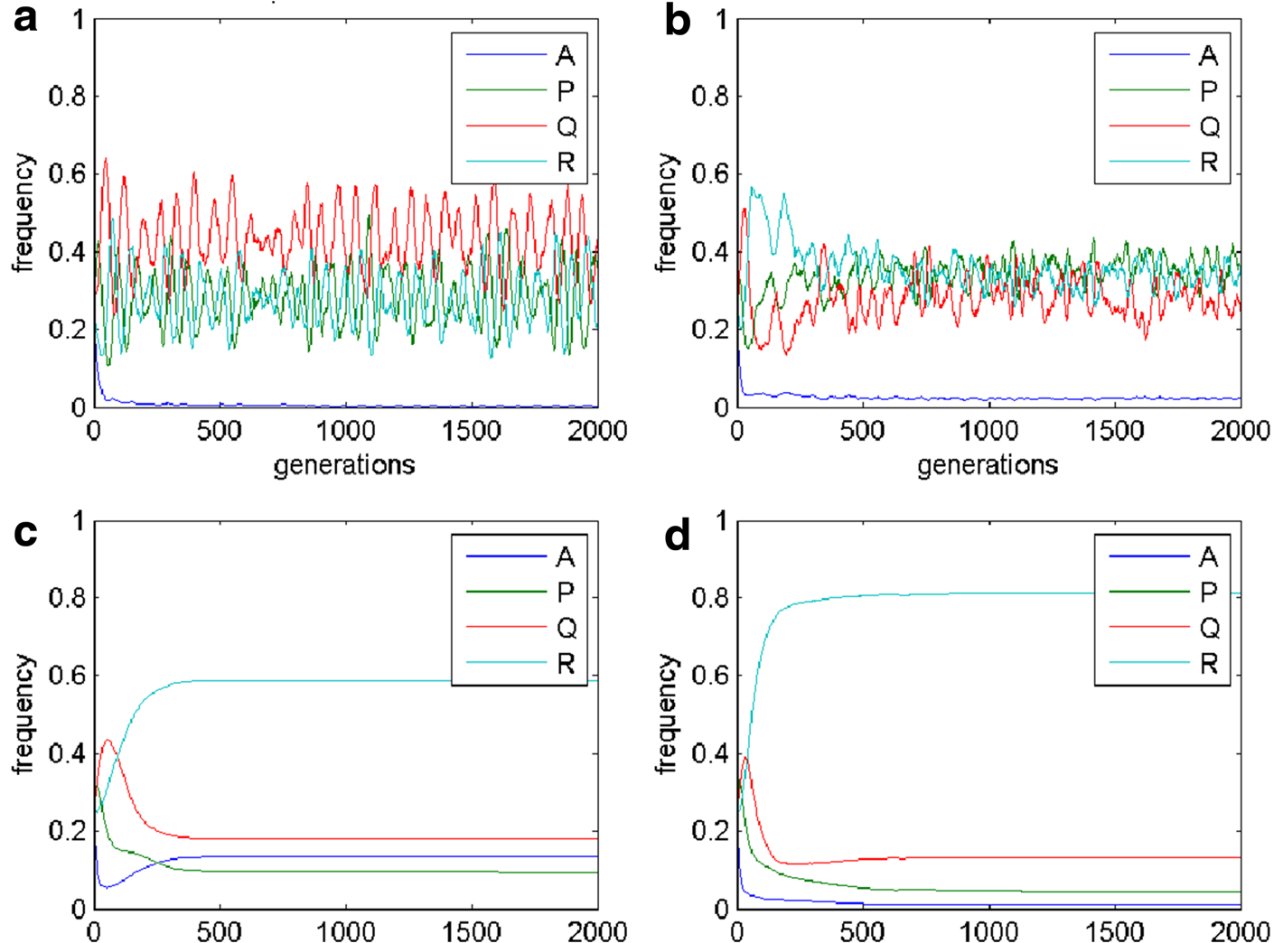
MSEG results (time chart) for i = 0.6, j = 0.4, f = 0.5, g = 0.5, e = 0.3, h = 0.1. **a** probabilistic: A = 0.01, P = 0.36, Q = 0.43, R = 0.20; **b** deterministic: A = 0.02, P = 0.36, Q = 0.26, R = 0.36; **c** weighted mean, best cells 3: A = 0.14, P = 0.09, Q = 0.18, R = 0.59; **d** weighted mean, intervals 5: A = 0.01, P = 0.05, Q = 0.13, R = 0.81

If the value of parameter g is increased, similarly as for the mean-field game, P cells are in majority. However, for the weighed mean from best players reproduction it is feasible that other phenotypes appear in the final population. In the case when i is greater than j (Figs. 9 and 10), A cells are repressed from the population (the same as for the mean-field games), while the frequencies of the occurrences for the rest phenotypes oscillate.

## Discussion

Recent works have focused on the evolutionary dynamics of tumours [24] and point out that factors important at the evolutionary level, like survival and proliferation, are the pivotal points in development of cancer as a heterogeneous population with different cells. Moreover, an additional key-factor (for game theory applications) has been studied mainly by Basanta and Anderson [13], which is the impact of the ecosystem or the interactions between tumour cells and their environment. They have already modelled changes in the cancer ecosystem in the context of different anti-cancer therapeutic strategies. Further development of spatial games may provide additional possibilities of simulating therapies by affecting different players (as elements on the spatial lattice) at a different level or even in a different way. The extension could be reached by additional simulation of the environment (or another factors affecting the cancer cells population) performed on the parallel lattice. The simulation could base on evolutionary game theory principles (for instance another MSEG game) or any different cellular automata rules or algorithms. Another spatial layer may be introduced due to changes in the phenotypic composition of the population that means changes in the basic game. Furthermore, a simulated phenomenon should be included in the payoff matrix. Thus, the approach provides the possibility to have different payoff matrices for each cell on the spatial lattice providing the asymmetry in the game. Basanta and Anderson [13] indicate that elimination of as many cancer cells as possible may not be essentially the best strategy. They found that destroying only some fraction of the cancer cells (with a particular phenotype) may be far more efficient. Additionally, influencing the way how cells interact with each other shall be also considered. Thus, using spatial games with additional simulations provides a possibility to study that conclusion using a vast amount of different configurations (especially for various initial lattices and simulated environments). Combinatory anticancer treatment that changes mentioned intercellular interactions (for instance by affecting environment) and eliminates only selected types and fractions of the cancer cells could be efficiently simulated using MSEG with additional simulation. The described therapy fits well to the so called evolutionary double bind model [25]. Presented model suggests using sequentially two different anticancer therapies that affect the payoff matrix in different ways for different phenotypes that are resistant to the treatments. In case of EGT and SEGT approaches, one phenotype is resistant to only one treatment at the time (or not resistant at all). By MSEG, one cell could be resistant partly to each treatment, which we find more akin to the biological reality. What is more, an additional lattice may be easily introduced to simulate the treatment concentration doses impacting the phenotypes by changing payoff matrix parameters.

The final lattice and abundance of particular phenotypes depend on the reproduction scheme (e.g. Fig. 8 phenotype P is dominant for the probabilistic reproduction, while phenotype R for the deterministic one). This confirms our expectations related to the role of the reproduction schemes (see, section Methods): the deterministic reproduction favors strongest players and the probabilistic one advantages social behaviors related to indirect interactions. Due to different payoff computing algorithms, the deterministic reproduction may describe and depend on the direct communication of the cells; on the other hand the probabilistic is related to bystander effect and its impact on the neighboring cells. Probabilistic and deterministic schemes allow for cell clustering and some stable results (Figs. 5 and 7), however, for a different set of payoff matrix values, some oscillations and changing cells structures may occur (Fig. 9). On the other hand, reproduction types based on weighted mean in all cases ‘smooth’ the lattice to the one type of phenotypes composition.

## Conclusion

In this paper spatial evolutionary games, proposed by Bach et al. [17] have been developed further following our proposal [14]. This new approach considers that each simple player shall be treated as a more complex individual expressing different traits, which seems to be more realistic for the simulation of many biological processes considering the heterogeneity of individuals. The proposed model is an extension of two of the first game theoretic models of carcinogenesis. The model assumes an existence of four possible phenotypes (strategies) in the population of cells that make up a tumour. One of the results is possibility of stable coexistence of different tumour cells within the population. Commonly known models describe single phenomenon (or single traits of the tumour), for instance, avoiding apoptosis [4], inducing angiogenesis [5] or activation of invasion or metastasis [18]. Some of them already cover hallmarks of the cancer presented by Hanahan and Weinberg [26, 27]. We believe that combing models (introducing more different phenotypes within one game) and introducing cells heterogeneity (by MSEG) provide possibility to study tumour cells evolutionary process [24]. Yet another possibility given by this approach is related to the potential of stem cells and their ability to behave differently depending on unknown environmental factors. In some sense they may play arbitrary strategy which in turn may lead to their differentiation. In [28] and [29] the authors see the benefits of applying the evolutionary game theory to modelling stem cells interaction with their environment and the surrounding cells. Studying a solution using EGT their models do not only describe the differentiation process of the stem cells, but also the heterogeneity of the cell population containing them. However, they do not consider the heterogeneity on the cells level, which could be introduced by MSEG.

The effects and potential interactions, both in macro and micro environments, may be better analysed and understood by spatial factors. To our knowledge, so far all comparisons with biological phenomena (in terms of game theoretic carcinogenesis modelling) have been performed only in a qualitative way which, still, may be very complicated in the case of more complex models. Moreover, we also emphasize strongly that evolutionary games are mainly used to study changes in a tumour’s phenotypic heterogeneity and its impact on the evolutionary dynamics of cancer (possibility of different interactions, e.g. cooperation). However, the importance of heterogeneity is at the population level, meaning that the population contains different homogenous cells, which is obviously an important limitation arising from the usage of replicator dynamics. The application of multilayer spatial evolutionary games additionally allows for modelling heterogeneity on the cell level within the population, which may be more appropriate for the biological reality.

Although the results of modelling and simulation have only quantitative meaning, they are biologically valid. Comparing them to results of different experiments on cell lines performed by biologists cooperating with us enables discussion of the impact of different parameters on the development of phenomena related to interactions of the cell populations. Our first attempt to mimic behaviour of real cell populations observed in such experiments using MSEG approach was successful and results of the modelling were presented in [30]. Moreover these results could be used to plan new experiments which may explain processes still far from being recognized. It also enables study of cancer as a network society of communicating smart cells [31].

A recent study [32] shows the possibility of training and validating the replicator dynamics equations using population sizes measured in co-culture over time, and the potential clinical implications discussed may enable future development and quantitative application of results from theoretical game models in cancer treatment. However, to apply fully the game theoretical models, it is necessary to find a way to train and validate the payoff matrices. That step would allow not only to simulate and validate scenarios where the numbers or frequencies of particular cells have been changed, but it would provide a way to study the changes within the interactions between cells (for instance by affecting the environment).

## Reviewers’ comments

First of all we would like to thank the reviewers for their valuable comments. We hope that the revision of the paper in which we have followed their remarks is now acceptable. In what follows, we detail the responses to more specific comments of the reviewers and changes introduced by us to the manuscript.

### Reviewer’s report 1: Tomasz Lipniacki

Reviewer comments:

The Authors propose approach to spatial cancer modeling based on evolutionary games on the lattice. They analyze competition between four cell phenotypes that can mimic various types of cells in the cancer subpopulations. The competition between these phenotypes is characterized by 6 parameters representing costs and gains in the game. The Authors show that depending to values of these parameters the systems may reach a different equilibrium in which one, two, three or four phenotypes coexists in the final population. Overall it is a nice study showing possible directions in heterogeneous cancer population modelling. I have some specific comments, addressing which may improve exposition of results and readability of the manuscript.

1. There is a long Methods section, but in addition a brief summary of simulations details would be helpful. For example the information about size of the lattice is missing.

Authors’ response: *We have added more detailed description of our simulation methodology in section Methods*.

2. The information that the problem is considered on 2D lattice should be given earlier, maybe in abstract.

Authors’ response: *This additional information has been included in the abstract*.

3. I am not sure whether the “multilayer spatial evolutionary game” is the right terminology, as the simulations are performed on single lattice (I think!) not on four lattices, and phenotypes densities sum to 1.

Authors’ response: *Additional explanation regarding the multiple layers has been added in section Methods. The lattice is 2D considering the cells neighbourhood, however from the computation point of view the game is played on as many lattices, or more precisely, as many layers of the lattice as is the number of pure strategies (basic phenotypes) in the pay-off table for a given game*.

4. Authors should discuss why the winning (or most abundant phenotype) depends on the model version, e.g. in Fig. 6 phenotype P is the most abundant for probabilistic model, while phenotype A is most abundant for remaining three models. The differences are also for parameters chosen to produce Figs. 8 and 10.

Authors’ response: *The variety of behaviours of cell populations depending on the choice of reproduction schemes and parameters results both from mathematical and biological reasons. In the conclusion and discussion sections we have added some comments on probable reasons of those differences, some of them could be expected from theoretical analysis, the others seem to be case specific or even paradoxical*.

5. Oscillations shown in Fig. 10 can be results of finite lattice size – please comment.

Authors’ response: *The lattice used in the simulations is a torus, thus it does not have the finite borders. However analysis of the results for different lattice sizes (bigger than used in the paper – 30×30) suggests that the appearance of the oscillations is not related to the size. It is rather dependent on the values of the payoff parameters.*

6. In Discussion and Conclusion Authors should refer more to their specific results shown in Figs. 1, 2, 3, 4, 5, 6, 7, 8, 9 and 10.

Authors’ response: *References to the specific results and figures have been added*.

7. Authors may consider adding some discussion about stemness and differentiation. Simulations in which cells can change their phenotype would be cool.

Authors’ response: *We are really grateful for this comment – we have found some interesting papers related to this problem which we refer to and comment in the revised version of the paper. We think that our approach may be especially valuable in tracking the fate of stem cells. The reviewer’s remark has inspired us to study this problem in our further research*.

### Reviewer’s report 2: Urszula Ledzewicz

Reviewer comments:

Originality: The authors propose a new type of spatial evolutionary games called multilayer spatial evolutionary games. The idea is that cells on a lattice are able to play a mixture of strategies instead of choosing one special strategy. In terms of phenotypes, which in evolutionary games are the strategies that the cells represent, different phenotypes with some degree of belongingness are used. Alternatively, there exists an almost continuous spectrum of phenotypes within the considered population of cells which combine basic traits observed in the population. Both these interpretations make biological sense and they may be a good description of cancer heterogeneity which is manifested not only on the population level but also at the cellular level. Such an approach has not been used before except for the previous paper of the authors [14] (numbers of references are as in the paper under review) in which, however, only the idea of mixing different phenotypes in the context of modeling a bystander effect is discussed without general rules and algorithms for its implementation. In this paper, the spatial game resulting from the interaction of cells representing phenotypes being mixtures of the basic traits is played on the lattice which contains as many layers as is the number of basic traits. This is another original contribution of this paper. An important advantage of this approach is that the number of phenotypes or traits represented by the cells is not critical for efficient computations. This is demonstrated in the paper where four different basic traits are discussed while in almost all papers in which evolutionary game theory has been used for modeling of tumor cells interactions only two or three phenotypes have been discussed. Moreover, thist leads to a new insight on the structure of the modeled cancer cell population. The model discussed in the paper combines two classical models of Tomlinson (presented in [4] and [5]) and such combination analyzed together seems to be important from the point of view of tumor growth and development. As mentioned before, multilayer evolutionary games enable modeling of almost a continuous spectrum of phenotypes. This “almost” results from a finite number of intervals representing contributions of different traits in the specific phenotype of the cell. This leads to yet another original idea proposed by the authors related to reproduction schemes used in the spatial evolutionary game algorithm. Two new such schemes are added to standard probabilistic and deterministic ones: mean value of best cells and mean value of best intervals. Although their biological interpretation is not evident, the results in the case when they are used seem to be compatible with mean field results. This procedure which is a kind of discretization is necessary because of the graphical interpretation of results. Since mixing phenotypes means mixing colors, feasibility of the analysis of the results depends on distinguishability of these colors. Significance: Heterogeneity of cells has become one of the most often discussed cancer hallmarks. Populations of living cells contain subpopulations which differ in phenotypes, and even cells that develop as clones from single cells show differences in cell cycle progression, production of specific proteins, or induction of processes leading to cell death after some time. The development of cell populations such as in a tumor depends on the phenotypic structure of the initial cell population and on the exchange of signals between cells via molecules released into the environment or placed on the cell surface. It has become clear that not only are distinct tumor subclones found to coexist within the same tumor regions, but that metastatic subclones originate from a non-metastatic parental clone in the primary tumor. Additional post-transcriptional and epigenetic changes can potentially further diversify a tumor population, which is also dynamic, as shown in the responses to standard regimens, with preexisting minor subclones expanding to dominate at relapse. Therefore, current regimens can have unpredictable and/or unintended consequences on the resulting tumor diversity. Current experimental approaches do not allow observations of single cells in a population for very long periods because of limitations such as nutrient depletion or overgrowth of cells, and studies of the molecular aspects of development in a cell population are more complex. Evolutionary game theory provides tools which help to understand the main processes that govern the development of structured cell populations. This type of analysis may help to understand differences of response to environmental or therapeutic factors between different cell types. The multilayer spatial evolutionary games proposed by the authors may explain results of many experiments in which, on the first view the same cancer cells in almost the same conditions behave differently. Moreover, the same approach may be used to study different effects of therapies treated as yet another player in the game. In light of recent studies showing the extent of intratumor heterogeneity and its clinical implications, it is important to incorporate tumor diversity and the expected evolutionary trajectories into rational drug design to achieve predictable tumor response, and reduce chances of relapse. Thus it might be preferable to employ a less radical treatment protocol that preserves heterogeneous therapeutically “naïve” population than to select for a very fast growing and resistant clone by using a “sledgehammer” therapy The multilayer approach could easily incorporate the effect of intervention and its mutual relationship with cancer heterogeneity. Nevertheless, the success of this technique is highly dependent on the possibility of estimation of parameters used in pay-off tables. Especially, as it is demonstrated in the paper, the results are very sensitive to these parameters. From one side the results obtained in the paper dealing with this sensitivity are important because they justify experimental results indication such sensitivity. On the other hand, taking into account difficulty in precise estimation of pay-off coefficients leads to the conclusion that the results of the proposed technique of modeling have only qualitative value. Moreover, new types of reproduction proposed in the paper open new possibilities of understanding some ‘altruistic’ behavior observed in some experimental studies on tumor cells. Unfortunately, all these prospective applications are not discussed in the paper. Such discussion may significantly improve its quality.

Authors’ response: *We wish to thank the reviewer for bringing to our attention some advantages of our approach. Frankly speaking, some of them has been “discovered” by us due to the reviewer’s comment. We have extended the discussion session to include some of them. On the other hand, just recently, our publication* [30] *prepared in collaboration with biologists from our institution, has appeared in which we reported our successful attempt to mimic results of biological experiment using MSEG.*

### Reviewer’s report 3: Jacek Banasiak

Reviewer comments:

Having read the paper carefully, I realized that I should not have accepted invitation to review it as evolutionary games is not my field of interest and also I am a mathematician and the appear does not contain much mathematics in the conventional style. Nevertheless, let me try to provide some comments. Evolutionary game theory has been used with some success to simulate tumour development. Spatial evolutionary games allows to model some spatial heterogeneity of cells. The main contribution of the paper is to extend the existing results of simulating tumour processes that have been limited to two or three phenotypes, to four phenotypes. Moreover, what the authors call mixed (or multilayer) spatial evolutionary games, allow each cell to play different strategy (out of these four). Different mixes of strategies are treated as different phenotypes. An important feature of the paper is bringing some parallel between the spatial evolutionary games and the replicator dynamics approach that looks at the ‘mean-field’ description of the game. According to the authors, extending the number of strategies to four, bringing the dimension of the mean-field model to three, allows the replicator dynamics (described by an ODE system) to exhibit more complex dynamics, including chaos (strange attractors). However, the authors have not pursued this comment. In general, the paper offers a description of the mixed spatial evolutionary game theory approach to cancer modelling in which not only heterogeneity in space but also at a given point, in the sense of possibility of having different phenotypes at any give site, can be modelled. This is illustrated by performing in two sets of simulations varying two out of four parameters in each one. Some comparison with results obtained by the mean-field approach for the same values of parameters as before.

There are some statements in the paper that should be re-considered.

- For instance, on p. 2, in Conclusions, the authors write: Despite complex analysis....., the model gives finite number of diverse results (meaning, I believe, few different results). On the other hand, on p. 16, line 35, they state: Due to immense amount of different results...., we discuss only the case when the population is quadromorphic. So, do we have just few different results, or an immense amount of them?
- The first sentence of the last paragraph on p. 7 would be more clear if a colon was used. The second sentence in that paragraph should be re-written { it is too convoluted to carry any meaning.
- Page 8, l. 29: if \every”, then the sentence should be in singular.
- Page 9, l. l. 8{10, at least semi-colon instead of comma, then I would write: this method allows for modelling situations that are biologically more realistic.
- Page 9, l. 21, invaded, I presume.
- Page 10, l. 16–17, the sentence should be somehow substantiated by e.g. referring to the simulation results. It is an important point as when one presents a new method that gives different results from a well-established one, some argument should be provided to convince the reader that the new method is better and why. Also, in the second sentence of this paragraph I would not use the verb ‘arise’. The construction of the sentence should be changed.
- Page 13, l. 15{, It is not clear what the paragraph is about, especially how the second sentence is related to the first.
- Page 13, l. 32, resulting model.
- Page 15, l. 26, ‘cons’ is a colloquial expression; after the comma, what is the meaning of ‘the exact ratio of phenotypes’ - something is missing.
- Page 16, l. 8, again, the authors state some fact without any attempt to reflect on it.
- Page 16, l. 28, if the parameter varies between 0 and 1, I would not say that the change by 0.1 (10 %) is small

Authors’ response: *We have done our best to make the revised version easier to understand. We hope that the English is much improved (a native English speaker has been involved in revision of the manuscript) and all typos and ambiguous sentences have been corrected*.

As I said earlier, this paper does not belong to the field I am comfortable doing reviews in. It is not a conventional mathematics. It offers a description of an interesting method of approaching the problem of modelling the evolution of spatial and local heterogeneity of cancer cells, together with some numerical simulations. Possibly the value of the paper would be improved if the simulations were tested against some real data.

Authors’ response: *The first attempt was already made by us and the results are reported in the paper* [30] *which we have added to the list of references*.

## Abbreviations

EGT: Evolutionary game theory
ESS: Evolutionary stable strategy
MSEG: Multilayer spatial evolutionary game
SEGT: Spatial evolutionary game theory
